# The effects of pre-treatment with olibanum and its constituent, boswellic acid on synaptic plasticity impairments induced by lipopolysaccharide in rats

**Published:** 2021

**Authors:** Narges Marefati, Farimah Beheshti, Sara Memarpour, Mohammad Rezaei, Mahmoud Hosseini

**Affiliations:** 1 *Neurogenic Inflammation Research Center and Department of Physiology, School of Medicine, Mashhad University of Medical Sciences, Iran*; 2 *Neuroscience Research Center, Torbat Heydariyeh University of Medical Sciences, Torbat Heydariyeh, Iran*; 3 *Department of Physiology, School of Paramedical Sciences, Torbat Heydariyeh University of Medical Sciences, Torbat Heydariyeh, Iran*; 4 *Mollecular Medicine Department, Mashhad University of Medical Sciences, Mashhad, Iran*; 5 *Student Research Committee, Department of Physiology, Faculty of Medicine, Mashhad University of Medical* *Sciences, Mashhad, Iran*; 6 *Division of Neurocognitive Sciences, Psychiatry and Behavioral Sciences Research Center, Mashhad University of Medical Sciences, Mashhad, Iran*

**Keywords:** Olibanum, Boswellic acid, Lipopolysaccharide, Synaptic plasticity, Inflammation, Oxidative stress

## Abstract

**Objective:**

Olibanum (OLIB) and its component boswellic acid (BOSA) are suggested to have anti-inflammatory, anti-oxidant and neuroprotective effects. In the present work, we examined effect of OLIB, and BOSA on the synaptic plasticity impairment and oxidative stress indicators in a rat model of neuro-inflammation induced by lipopolysaccharide (LPS).

**Materials and Methods:**

Forty rats were divided into the following four groups: (1) Control, (2) LPS, (3) OLIB (200 mg/kg), and (4) BOSA (10 mg/kg). The animals were pre-treated with OLIB extract, BOSA or the vehicle 30 min before LPS (1 mg/kg) administration, for 6 days. On the 6^th^ day, electrophysiological recording was done. Long-term potentiation (LTP) from CA1 area of hippocampus was assessed. The animals were then sacrificed and their brains were removed for evaluation of the levels of interleukin-6 (IL-6), nitric oxide (NO) metabolites, malondialdehyde (MDA), thiol, superoxide dismutase (SOD) and catalase (CAT) in the cortex.

**Results:**

Administration of LPS decreased amplitude (p<0.001) and slope (p<0.01) of field excitatory postsynaptic potential (fEPSP). Pre-treatment enhanced these parameters (p<0.05 to p<0.001). LPS also increased cortical levels of IL-6 (P<0.01), NO, and MDA (p<0.001) while decreased thiol, SOD (p<0.001), and CAT (p<0.05). OLIB and BOSA diminished IL-6 (p<0.05-p<0.001), NO (p<0.01-p<0.001) and MDA level (p<0.01 and p<0.001, respectively) while improved SOD (p<0.05 and p<0.001, respectively), CAT (p<0.05 and p<0.001, respectively) and thiol content (p<0.001).

**Conclusion:**

The results showed that OLIB and BOSA could improve synaptic plasticity impairment induced by LPS as shown by a decrease in an inflammation indicator along with the anti-oxidant effects.

## Introduction

Inflammation is a complex process caused by disruptive factors such as infection and chemical damage (Ashley et al., 2012). During inflammatory responses, the release of inflammatory cytokines including interleukin-1 beta (IL-1β), IL-6 and tumor necrosis factor alpha (TNF-α) is increased in the brain. These inflammatory cytokines directly affect neuronal functions including long-term potentiation (LTP), glutamate release, AMPA receptor density, and cellular signaling pathways involved in memory (Czerniawski et al., 2015).There is also evidence that oxidative stress and reactive oxygen species (ROS) impair memory and learning (Behl and Moosmann, 2002). Studies have also reported that lipid peroxidation induced by oxidative stress, affects memory and learning performance in rats (Abidin et al., 2004). Lipopolysaccharide (LPS) is a Gram-negative bacterial endotoxin (Dantzer et al., 2008) that, according to numerous studies, enhances the synthesis and release of inflammatory cytokines such as TNF-α, IL-1β and IL-6, and induces oxidative stress in the central nervous system (Dehmer et al., 2004) which impairs synaptic communication, and disrupts LTP, resulting in learning and memory impairment (Hritcu et al., 2011; Saavedra, 2012). 

The resin of *Boswellia serrata*, from the Burseraceae family, is obtained from the bark of the tree, commonly known as Frankincense or Olibanum (OLIB) (Kimmatkar et al., 2003). It was noticed in many studies that this herbal compound has a beneficial effect on treatment of inflammatory diseases such as rheumatoid arthritis (Etzel, 1996), inflammatory bowel disease, bronchial asthma (Qurishi et al., 2010), osteoarthritis (Kimmatkar et al., 2003), and chronic colitis (Gupta et al., 2001). More than 200 constituents known to be present in OLIB, were shown to be responsible for therapeutic properties against inflammatory diseases (Camarda et al., 2007; Etzel, 1996). The presence of triterpenoid, such as pentacyclic triterpenoid known as boswellic acid (BOSA) in OLIB, leads to indication of anti-inflammatory effects (Kumar et al., 2012). OLIB inhibits inflammation in many inflammatory diseases by specifically inhibiting 5-lipooxygenase enzyme, preventing leukotriene synthesis and stopping glycosaminoglycan production (Weber et al., 2006). According to some evidence obtained by animal studies, OLIB and its constituent, BOSA have obvious positive effects on neuro-inflammation diseases (Hosseini et al., 2010; Weber et al., 2006). It was also suggested that OLIB and its active ingredient BOSA, could be useful in improving a variety of models of cognitive dysfunction and memory impairment caused by hypothyroidism (Hosseini et al., 2010), diabetes (Gomaa et al., 2019), seizure (Jalili et al., 2014), Alzheimer's disease (Beheshti et al., 2016) and stroke (Jivad et al., 2015). To the best of our knowledge, there is no study that elucidated the effect of OLIB and BOSA on synaptic dysfunction induced by LPS. Therefore, the present study was aimed to investigate the effects of OLIB and BOSA on synaptic dysfunction, IL-6 concentration and oxidative stress indicators in the brain, in a rat model of neuro-inflammation induced by LPS.

## Materials and Methods

**Study design**

Forty rats in the weight range of 200-250 g, were used in this experiment. The animals were kept under a 12-hour light/darkness cycle in the Animal House of Mashhad University of Medical Sciences at the optimized temperature of 23±2^o^C and they had free access to food and drinking water. The Ethical Committee of Mashhad University of Medical Sciences approved the study (NO: IR.MUMS.fm.REC.1396.713). Animals were divided into 4 groups, as follows:

1) Control group: the treatments included normal saline (1 ml/kg body weight, i.p.) instead of LPS and DMSO diluted (%10) by saline (i.p.) instead of OLIB and BOSA, given for 6 days. 

2) LPS group: LPS (1 mg/kg body weight, i.p.) (Hakimi et al., 2019) was injected and DMSO diluted (%10) in saline was administered instead of OLIB and BOSA during 6 days.

3) OLIB group: OLIB (200 mg/kg body weight, i.p.) (Zaker et al., 2015) was administered and 30 min later, LPS was daily injected during 6 days.

4) BOSA group: BOSA (10 mg/kg body weight, i.p.) (Sayed and El Sayed, 2016) was administered and 30 min later, LPS was daily injected during 6 days.

On the 6^th^ day, the electrophysiological recording was performed two hours after LPS injection. After electrophysiology recording, the animals were sacrificed under a deep anesthesia provided by urethane. The brains were removed and stored at -80^o^C for evaluation of IL-6, nitric oxide (NO) metabolites, malondialdehyde (MDA), thiol, superoxide dismutase (SOD) and catalase (CAT) levels.

**Preparation of the extract and active ingredient**

The dried OLIB was purchased from a herbal drug store and approved by the Pharmacology Department of Mashhad University of Medical Sciences. Fifty grams of the grinded resin was mixed with 200 ml of ethyl acetate and placed for 24 hr at 37^ o^C inside a shaker-incubator. Then, the resulting mixture was placed into a solvent removal apparatus to yield the extract. In addition, BOSA was purchased from Tinab Shimi Khavarmianeh Company. Both OILB extract and BOSA were dissolved in dimethyl sulfoxide (DMSO) and diluted with saline (final concentration %10). 

**Electrophysiological study**

To perform electrophysiological assessments, the animals from all groups were anesthetized using urethane (1.6 g/kg body weight, i.p.), then, the head was fixed by the ear bars in the stereotaxic instrument. Considering bregma as a reference point and the coordinates obtained from the atlas of Paxinos, the CA1 location and Schaffer collateral pathway were determined. Then, two small holes were made on them under sterile condition. In order to record post-synaptic excitatory potentials, a dipole excitatory electrode was placed onto the right Schaffer collateral pathway. There was also a unipolar stability electrode fixed on the right CA1 area. The electrodes were then connected to the electric measuring instrument. If the field excitatory post-synaptic potential (fEPSP) waves were observed, the electrodes were adjusted to record the waves with constant amplitude and slope. Then, the animals were given a rest for 30 min. Following the stability of recording, the protocol of excitation/response was applied to assess the synaptic capacity before the long-term potentiation (LTP) induction. Following determination of a maximum response, a basic recording was performed for 30 min before inducing LTP. Then, high frequency stimulation (HFS, 100 Hz) was applied to induce LTP. The recoding was then followed for 90 min. The stereotaxic and physiological signs were assessed in order to make sure the electrodes were fixed at a correct point. For the physiological confirmation, the paired pulse (PP) 50 protocol, a pair of excitations with a 50-millisecond interval, was applied. In this status, the higher amplitude of the first wave in comparison to the second one, was indicative of the correct place of the electrodes. Finally, in electrophysiology analysis, the percentage of amplitude and slope in all groups were compared (Abareshi et al., 2016).

**Measurement of the IL-6, NO metabolites and MDA concentrations**

The cortical level of IL-6 was determined based on the ELISA kit instructions (IBL International, Hamburg Germany). In ELISA technique, antibodies labeled with enzyme are used to identify antigens. After performing the corresponding protocol, according to absorbance which was read in a 96-well microplate reader (Biotech, USA) and plotted standard curve, the IL-6 level was calculated. To estimate the NO metabolites concentration, the Griess kit (Promega Company) was used and the protocol provided in the kit, was followed. To measure MDA, 1 ml of 10% homogenate tissue solution was mixed with 2 ml of thiobarbituric acid (TBA), trichloroacetic acid (TCA) and hydrochloric acid (HCL) solution and heated for 45 min in a boiling water bath. After cooling to room temperature, it was centrifuged at 1000 rpm for 10 min. Then, the absorbance was read at 535 nm and MDA concentration was calculated based on the corresponding formula (Quan et al., 1994).

**Measurement of SOD and CAT activities and thiol content**

Enzymatic activity of SOD was measured by Madesh and Balasurbamanian colorimetric method using 96-well plates at 570 nm (Madesh and Balasubramanian, 1998; Marefati et al., 2020). The activity of CAT was measured based on its ability to decompose hydrogen peroxide (H_2_O_2_), using Aebi method (Aebi, 1984).

To measure thiol content, 1 ml homogenate sample was added from ethylenediaminetetraacetic acid (EDTA) buffer (30 mM tris), and 3 mM EDTA (pH 8.2) and the absorbance was read at 412 nm against tris-EDTA buffer alone. Then, 20 µl of 10 mM dithionitrobenzoic acid (DTNB) reagent (in methanol) was added and the sample was re-read after 15 min at room temperature. The adsorption of DTNB solution was also read as a blank and the total thiol content (mM) was calculated using the corresponding formula (Koppenhöfer et al., 2015):

(∆A570/min uninhibited - ∆A570/min inhibited) ÷ (∆A570/min uninhibited - ∆A570/min blank) = Inhibition (%)

 Enzyme activity (unit/ml) = Inhibition (%) ÷ 50% × sample

**Statistical analysis**

The data is shown as mean±standard error of mean (SEM). Data was analyzed by SPSS 16 software. One-way ANOVA statistical tests followed by Tukey *post hoc *test was used for analyzing biochemical data. The amplitude and slope of fEPSP in the electrophysiological evaluations was analyzed using repeated measures ANOVA statistical test followed by Tukey *post hoc *test. The differences were considered significant when p<0.05.

## Results

**Electrophysiological results**

**Figure 1:**
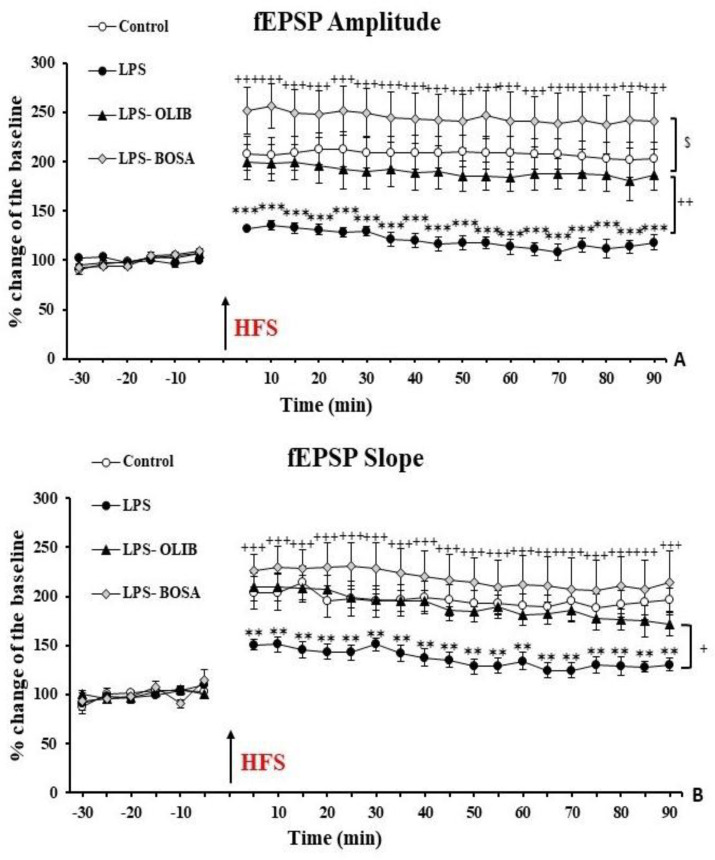
The results of LTP induction in the CA1 area of hippocampus. The amplitude (A) and slope (B) of fEPSPs are indicated as percentage among the groups. Data is presented as mean±SEM. **p<0.01 and ***p<0.001 represent the difference compared to the control group. ^+^p<0.05, ^++^p<0.01, and ^+++^p<0.001 represent the difference compared to the LPS group. ^$^p<0.05 represents the difference compared to the OLIB group

According to the results, it was demonstrated that following high frequency excitations, the amplitude of fEPSPs in the LPS group was significantly lower than that of the control group (p<0.001). On the other hand, administration of both OLIB and BOSA led to a considerable increase in fEPSPs amplitude compared to the LPS group (p<0.01 and p<0.001, respectively). The results also showed that amplitude of fEPSPs in the BOSA group, was higher than the OLIB group (p<0.05). In addition, no significant difference was seen between the OLIB and BOSA groups compared to the control group (Figure 1A).

As it is shown in Figure 1B, the results indicated that the fEPSP slope significantly decreased in the LPS group compared to the control (p<0.01), while administrations of OLIB (p<0.05) and BOSA (p<0.001) were accompanied by a meaningful increase in fEPSP slope compared to the LPS-treated animals. There was no significant difference between OLIB and BOSA groups. Also, no significant difference was seen between the OLIB and BOSA groups compared to the control group (Figure 1B).

**Biochemical results**

**Both OLIB and BOSA decreased IL-6, NO and MDA levels**

According to the results, the cortical IL-6 levels in the LPS group increased significantly compared to the control animals (p<0.01), whereas in both OLIB (p<0.05) and BOSA (p<0.001) groups, it was lower than that of the LPS group. 

**Figure 2:**
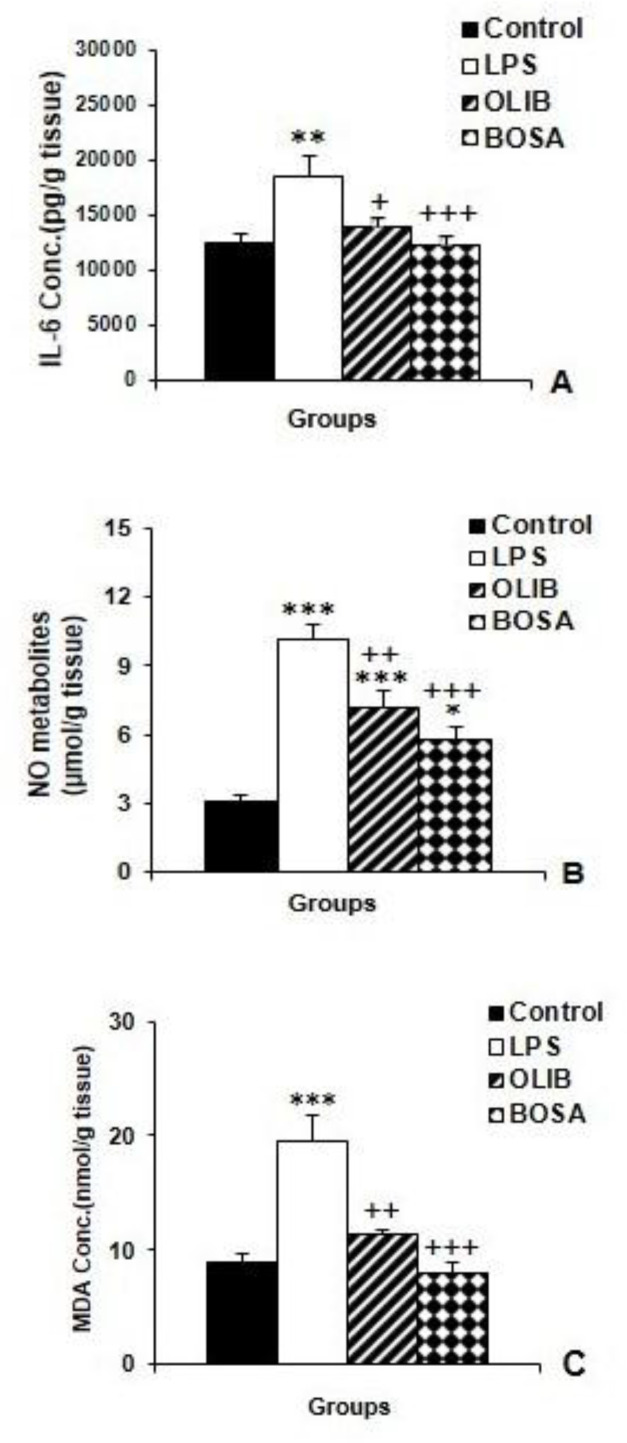
The comparison of cortical IL-6 (A), NO metabolites (A) and MDA levels among the groups. Data is presented as mean±SEM. *p<0.05, **p<0.01, and ***p<0.001 represent the difference compared to the control group. ^+^p<0.05 and ^++^p<0.01, and^ +++^p<0.001 represent the difference compared to the LPS group

The results also showed that there was no significant difference in IL-6 level between BOSA and OLIB groups (Figure 2A). Moreover, LPS injection was accompanied by an increased level of NO metabolites in the brain (p<0.001). Pre-treatment with both OLIB (p<0.01) and BOSA (p<0.001) decreased NO metabolites. In the brain of both OLIB and BOSA groups, the NO metabolites concentration was still higher compared to the control group (p<0.001 and p<0.05 respectively); while, there was no significant difference between OLIB and BOSA groups (Figure 2B).

Biochemical measurements also showed that MDA as an index of lipid peroxidation, was increased in the brain following LPS injection (p<0.001). Administration of both OLIB (p<0.01) and BOSA (p<0.001) decreased MDA in the brain compared to the LPS group (Figure 2C). With respect to MDA levels, there was no significant difference between OLIB and BOSA groups in compare to the control group. Also, there was also no significant difference between OLIB and BOSA groups (Figure 2C).

**Both OLIB and BOSA improved SOD, CAT and thiol levels**

**Figure 3:**
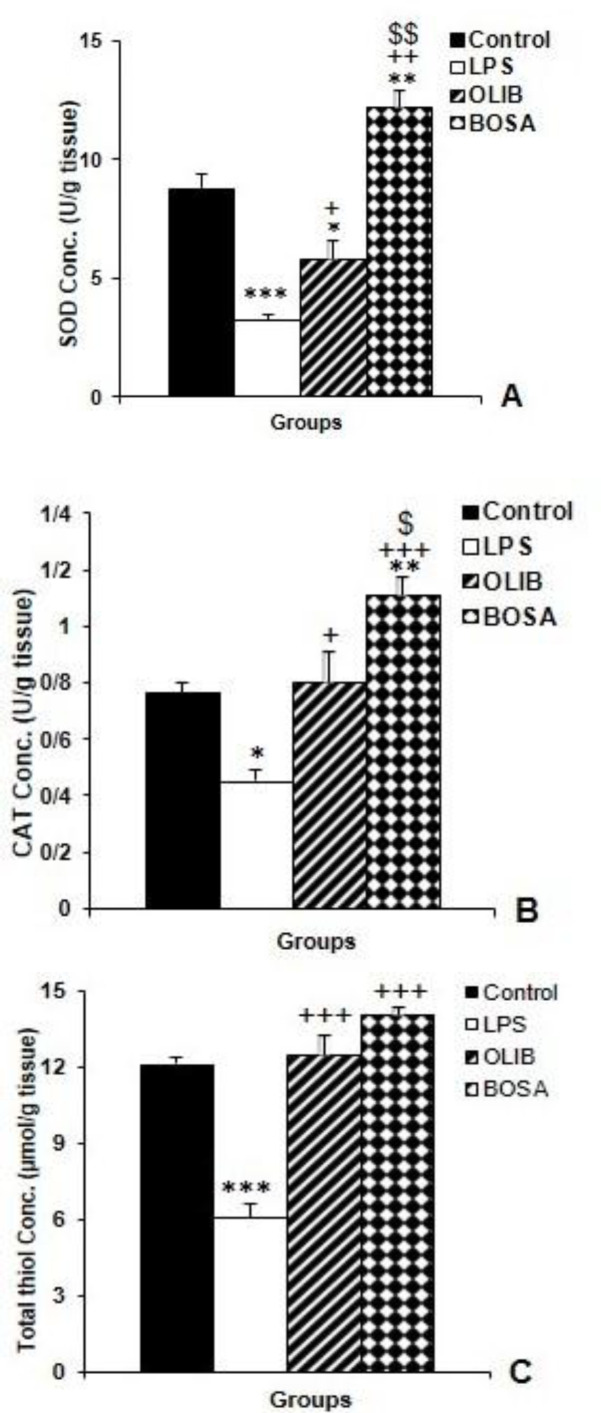
The comparison of cortical SOD (A), CAT (B) and total thiol groups (C) levels among the groups. Data is presented as mean±SEM. *p<0.05, **p<0.01, and ***p<0.001 represent the difference compared to the control group, ^+^p<0.05 and ^+++^p<0.001 represent the difference compared to the LPS group. ^$^p<0.05 represents the difference compared to the OLIB group

Based on our results (Figures 3A), LPS administration led to a significant decrease in cortical SOD (p<0.001) compared to the LPS group, but both OLIB and BOSA increased SOD activity (p<0.05 and p<0.001, receptively). In addition, SOD activity in the brain of the BOSA group was higher (p<0.001) while in OLIB group was lower (p<0.05) than the control group. The results also showed that SOD activity in the brain of BOSA group was higher than that of the OLIB group (p<0.001). 

 In addition, Figure 3B shows that, LPS diminished the cortical CAT activity compared to the control group (p<0.05) while, pre-treatments with OLIB (p<0.05) and BOSA (p<0.001) elevated this parameter. Brain tissue CAT activity in the BOSA group, was higher than that in the control (p<0.01) and OLIB (p<0.05) groups (Figure 3B). 

Concerning biochemical data, it was found that LPS injection was associated with a decrease in thiol content (p<0.001) but pre-treatments with OLIB and BOSA increased thiol levels compared to the LPS group (p<0.001 for both cases). There was no significant difference between the OLIB and BOSA groups in compare to control group. There was no significant difference between OLIB and BOSA groups (Figure 3C). 

## Discussion

The present study focused on the effect of administration of OLIB and its constituent BOSA, on LTP impairment due to neuro-inflammation induced by LPS. The results of the current study showed a significant positive effects of OLIB and BOSA on recovery of synaptic plasticity impairment which was induced by administration of LPS, as documented by reduction of inflammation cytokine IL-6, weakening of oxidative stress parameters including NO and MDA and strengthening of the anti-oxidant system components including SOD, CAT and thiol, in the brain.

LPS is a component of the outer membrane of Gram-negative bacteria, which has been used in many laboratory studies to induce inflammation models (Beheshti et al., 2019). LPS injection increases the production of inflammatory cytokines including TNFα, IL-1β, IL-6, prostaglandin E2 (PGE2) and interferon-gamma (IFN-γ) by microglia cells in the brain (Hakimi et al., 2019). In the current study, LPS injection was followed by increased levels of IL-6 in the brain which confirms neuro-inflammation status induced by LPS. 

Additionally, reactive oxygen species (ROS) were shown to be involved in the mechanism of toxicity induced by LPS, particularly by activating the nuclear factor kappa (NFκ) B transcription factor (Dehmer et al., 2004). Previous studies demonstrated that LPS injection increases the level of MDA while, decreases the glutathione level (Kheir-Eldin et al., 2001) and CAT and SOD activity (Kacem et al., 2015). The results of the current research also showed that MDA increased while thiol content and the activities of CAT and SOD decreased which confirms that LPS injection was followed by an oxidative stress status. 

Long-term Potentiation (LTP), known as a cellular model of synaptic plasticity, reflects a long-term increase in synaptic efficiency and it is an essential component of memory formation (Abareshi et al., 2016; Greenstein et al., 1988). To induce LTP in the CA1 region of the hippocampus, activation of glutamate N-Methyl- d-aspartate (NMDA) receptors is required in order to induce calcium and cascade activity of intracellular enzymatic events that affect different forms of synaptic plasticity (Connor et al., 1999). Scientific evidence suggests that LPS impairs synaptic plasticity and LTP (Anaeigoudari et al., 2015) induction in the CA1 region of the hippocampus by possibly reducing calcium entry via glutamate NMDA receptors (Jo et al., 2001). Another possible mechanism of LPS effect on LTP is the induction of inflammatory cytokines which directly impair neuronal functions such as release of neurotransmitter including glutamate, decrease density of the α-amino-3-hydroxy-5-methyl-4isoxazolepropionic acid (AMPA) receptors and suppress the cellular signaling pathways involved in LTP and finally impair memory performance (Czerniawski et al., 2015). Studies demonstrated that increasing the production of IL-1β followed by neuro-inflammation, leads to reduced neurotrophic factors including brain-derived neurotrophic factor (BDNF) production (Podda et al., 2016) which is well known to have beneficial effects on synaptic plasticity and memory (Zheng and Quirion, 2004). According to the results of the present study, the amplitude and slope of fEPSP in the animals of the LPS group were significantly reduced compared to the control group. 

Moreover, in our study, the levels of IL-6 and MDA in the LPS group were significantly increased compared to the control group, whereas the concentrations of SOD, CAT and total thiol groups in cortical tissue decreased in the LPS group. Probably, these results showed that, LPS injection resulted in impairment of synaptic plasticity and induction of LTP through induction of inflammation, strengthening of oxidant system and weakening of the antioxidant enzymatic system mediation. 

From ancient to the present time, various herbs have been cultivated for therapeutic purposes and used in traditional medicine. The use of these herbs seems to be the basis of modern medicine and many herbal remedies (Bansal et al., 2013). From the past to the now, *Boswellia serrata* from the Burceracea family has been used for various therapeutic purposes, especially in prevention and improvement of inflammation diseases (Weber et al., 2006). The anti-inflammatory therapeutic effects of *Boswellia serrata* are related to the resin OLIB and effective compounds of this resin which are more than 200 constituents such as BOSA and incensole acetate (Moussaieff and Mechoulam, 2009). So, the use of the extract of OLIB and the main component of the resin OLIB, was considered treatments in this study. In our experiment, both the OLIB extract and BOSA effectively decreased IL-6 level as a marker of neuro-inflammation. The effects were also associated with suppression of lipid peroxidation presented by a decrease in MDA concentration and increase of antioxidant enzymatic mediators. These results confirm the anti- inflammatory and anti-oxidant effects of both OLIB and BOSA. According to previous studies, the anti-inflammatory properties of OLIB are due to inhibition of the 5- lipoxygenase enzyme which is the key enzyme in biosynthesis of leukotriene (Upaganlawar and Ghule, 2009). According to other reports, OLIB inhibits expression of inflammatory cytokines like IL-6, IL-1 and TNF-α in parkinsonian rats (Ameen et al., 2017). Also, previous studies on the effects of OLIB on the central nervous system showed that it had a positive effect on growth of dendritic branches, augmented volume of hippocampal pyramidal layer and reduced the speed of changes to dendrite win analysis (Hosseini-sharifabad and Esfandiari, 2015). In the current research it was shown that both OLIB and BOSA improved synaptic function in the brain as presented by amplification of amplitude and slope of f EPSP. Considering these results, synaptic function improving effects of OLIB and BOSA seem to be due to their anti-inflammatory and anti-oxidant effects. 

Similar to our results, it was previously mentioned that OLIB inhibited the oxidative stress through increasing glutathione content alongside reducing theMDA level in comparison to the trimethyltin-administered group and improved learning and memory (Ebrahimpour et al., 2017). According to the results of another study, *B. serrata* gum extract reduced the hippocampal levels of caspase-3, cholinesterase (ChE), TNF-α, IL-1β, IL-6, and MDA levels in diabetic rats. Also, the extract increased the hippocampal level of glutathione (GSH), SOD and glutamate receptor expression and prevented from cognitive impairment and insulin resistance in diabetic rats (Gomaa et al., 2019).

It was repeatedly reported that NO has both positive and negative effects on learning, memory and LTP. At a physiologic level, NO improves cognition, learning, memory and synaptic function (Dubey et al., 2019; Ivanova et al., 2020) while it has an adverse effect when it is overproduced (Marottoli et al., 2017). It was repeatedly reported that high levels of NO were produced during neuro-inflammation to take part in brain tissues oxidative damage and play a negative role in learning, memory and synaptic function (Beheshti et al., 2019). On the other hand, inhibition of NO production was reported to be followed by a reduced level of neuro-inflammation and brain tissues oxidative damage (Beheshti et al., 2019). In the current study, positive effects of both OLIB and BOSA on LTP were accompanied by a decrease in NO metabolites in the brain and seem to have a role in the beneficial effects seen in the current research. 

In the current research, synaptic function improving effects of BOSA were greater than OLIB. The results also showed that BOSA was more effective in improving SOD and CAT activities but no significant difference was found between the two treatments in terms of MDA, IL-6, NO and thiol concentrations. It is mentionable that the dose of OLIB which was used in the current research was much higher than BOSA. Considering these results it seems that the beneficial effects of OLIB extract seen in the present work, are related to its component BOSA; however, more investigations are required in this context. 

OLIB because of its active ingredient BOSA, showed anti-inflammatory and anti-oxidant properties and improved synaptic function in a rat model of neuro-inflammation due to LPS administration. 

## References

[1] Abareshi A, Anaeigoudari A, Norouzi F, Shafei MN, Boskabady MH, Khazaei M, Hosseini M (2016). Lipopolysaccharide-induced spatial memory and synaptic plasticity impairment is preventable by captopril. Avd Med Sci-Poland.

[2] Abidin I, Yargicoglu P, Agar A, GÜMÜSLÜ S, Aydin S, Aydin S, ÖZTÜRK O, Sahin E (2004). The effect of chronic restraint stress on spatial learning and memory: relation to oxidant stress. Int J Neurosci.

[3] Aebi H (1984). Catalase in vitro Methods in enzymology.

[4] Ameen AM, Elkazaz AY, Mohammad HM, Barakat BM (2017). Anti-inflammatory and neuroprotective activity of boswellic acids in rotenone parkinsonian rats. CAN J Physiol Pharma.

[5] Anaeigoudari A, Shafei MN, Soukhtanloo M, Sadeghnia HR, Reisi P, Nosratabadi R, Behradnia S, Hosseini M (2015). The effects of L-arginine on spatial memory and synaptic plasticity impairments induced by lipopolysaccharide. Adv Biomed Res.

[6] Ashley NT, Weil ZM, Nelson RJ Bansal N, Mehan S, Kalra S, Khanna D (2013). Boswellia serrata-frankincense (A Jesus Gifted Herb); an updated pharmacological profile. Pharmacologia.

[7] eheshti F, Hosseini M, Hashemzehi M, Soukhtanloo M, Khazaei M, Shafei MN (2019). The effects of PPAR-gamma agonist pioglitazone on hippocampal cytokines, brain-derived neurotrophic factor, memory impairment, and oxidative stress status in lipopolysaccharide-treated rats. Iran J Basic Med Sci.

[8] Beheshti S, Aghaie R (2016). Therapeutic effect of frankincense in a rat model of Alzheimer’s disease. AJP.

[9] Behl C, Moosmann B (2002). Antioxidant neuroprotection in Alzheimer's disease as preventive and therapeutic approach. Free Radic Biol Med.

[10] Camarda L, Dayton T, Di Stefano V, Pitonzo R, Schillaci D (2007). Chemical composition and antimicrobial activity of some oleogum resin essential oils from Boswellia spp (Burseraceae). Annali di Chimica: Env Cult Heart Chem.

[11] Connor JA, Petrozzino J, Pozzo-Miller LD, Otani S (1999). Calcium signals in long-term potentiation and long-term depression. CAN J Physiol Pharma.

[12] Czerniawski J, Miyashita T, Lewandowski G, Guzowski JF (2015). Systemic lipopolysaccharide administration impairs retrieval of context–object discrimination, but not spatial, memory: evidence for selective disruption of specific hippocampus-dependent memory functions during acute neuroinflammation. Brain Behav Immun.

[13] Dantzer R, O'Connor JC, Freund GG, Johnson RW, Kelley KW (2008). From inflammation to sickness and depression: when the immune system subjugates the brain. Nat Rev Neurosci.

[14] Dehmer T, Heneka MT, Sastre M, Dichgans J, Schulz JB (2004). Protection by pioglitazone in the MPTP model of Parkinson's disease correlates with IκBα induction and block of NFκB and iNOS activation. J Neurochem.

[15] Dubey H, Gulati K, Ray A (2019). Alzheimer's disease: A contextual link with nitric oxide synthase. Curr Mol Med.

[16] Ebrahimpour S, Fazeli M, Mehri S, Taherianfard M, Hosseinzadeh H (2017). Boswellic acid improves cognitive function in a rat model through its antioxidant activity:-neuroprotective effect of boswellic acid. J Pharmacopunct.

[17] Etzel R (1996). Special extract of Boswellia serrata (H 15) in the treatment of rheumatoid arthritis. Pytoemy.

[18] Gomaa AA, Makboul RM, Al-Mokhtar MA, Nicola MA (2019). Polyphenol-rich Boswellia serrata gum prevents cognitive impairment and insulin resistance of diabetic rats through inhibition of GSK3β activity, oxidative stress and pro-inflammatory cytokines. Biomed Pharmacother.

[19] Greenstein YJ, Pavlides C, Winson J (1988). Lonf-term potentiation in the dentate gyrus is preferentially induced at theta rhythm periodicity. Brain Res.

[20] Gupta I, Parihar A, Malhotra P, Gupta S, Lüdtke R, Safayhi H, Ammon HP (2001). Effects of gum resin of Boswellia serrata in patients with chronic colitis. Planta Medica.

[21] Hakimi Z, Salmani H, Marefati N, Arab Z, Gholamnezhad Z, Beheshti F, Shafei MN, Hosseini M (2019). Protective Effects of Carvacrol on Brain Tissue Inflammation and Oxidative Stress as well as Learning and Memory in Lipopolysaccharide-Challenged Rats. Neurotox Res.

[22] Hosseini-sharifabad M, Esfandiari E (2015). Effect of Boswellia serrata gum resin on the morphology of hippocampal CA1 pyramidal cells in aged rat. Anat Sci Int.

[23] Hosseini M, Hadjzadeh MAR, Derakhshan M, Havakhah S, Rassouli FB, Rakhshandeh H, Saffarzadeh F (2010). The beneficial effects of olibanum on memory deficit induced by hypothyroidism in adult rats tested in Morris water maze. Arch Pharm Res.

[24] Hritcu L, Ciobica A, Stefan M, Mihasan M, Palamiuc L, Nabeshima T (2011). Spatial memory deficits and oxidative stress damage following exposure to lipopolysaccharide in a rodent model of Parkinson's disease. J Neurosci Res.

[25] Ivanova VO, Balaban PM, Bal NV (2020). Modulation of AMPA Receptors by Nitric Oxide in Nerve Cells. Int J Mol Sci.

[26] Jalili C, Salahshoor M, Pourmotabbed A, Moradi S, Roshankhah S, Darehdori A S, Motaghi M (2014). The effects of aqueous extract of Boswellia Serrata on hippocampal region CA1 and learning deficit in kindled rats. Res Pharm Sci.

[27] Jivad N, Rafieian-Kopaei M, Rezaei-Kheirabadi F, Khosravi S, Azizi M (2015). A study of the clinical efficacy of frankincense in the acute phase of ischemic stroke. Adv Herb Med.

[28] Jo J-H, Park E-J, Lee J-K, Jung M-W, Lee C-J (2001). Lipopolysaccharide inhibits induction of long-term potentiation and depression in the rat hippocampal CA1 area. Eur J Pharmacol.

[29] Kacem M, Simon G, Leschiera R, Misery L, ElFeki A, Lebonvallet N (2015). Antioxidant and anti-inflammatory effects of Ruta chalepensis L extracts on LPS-stimulated RAW 264 7 cells. In Vitro Cell Dev Biol Anim.

[30] Kheir-Eldin AA, Motawi TK, Gad MZ, Abd-ElGawad HM (2001). Protective effect of vitamin E, β-carotene and N-acetylcysteine from the brain oxidative stress induced in rats by lipopolysaccharide. Int J Biochem.

[31] Kimmatkar N, Thawani V, Hingorani L, Khiyani R (2003). Efficacy and tolerability of Boswellia serrata extract in treatment of osteoarthritis of knee–a randomized double blind placebo controlled trial. Phytomedicine.

[32] Koppenhöfer D, Kettenbaum F, Susloparova A, Law J, Vu X, Schwab T, Schäfer K, Ingebrandt S (2015). Neurodegeneration through oxidative stress: Monitoring hydrogen peroxide induced apoptosis in primary cells from the subventricular zone of BALB/c mice using field-effect transistors. Biosens Bioelectron.

[33] Kumar S, Prasad A, Iyer VS, Sahu A (2012). Systemic review: Pharmacognosy, Phytochemistry and Pharmacology of Martynia annua. Int J Res Med.

[34] Madesh M, Balasubramanian K (1998). Microtiter plate assay for superoxide dismutase using MTT reduction by superoxide. Indian J Clin Biochem.

[35] Marefati N, Beheshti F, Memarpour S, Bayat R, Shafei MN, Sadeghnia HR, Ghazavi H, Hosseini M (2020). The effects of acetyl-11-keto-β-boswellic acid on brain cytokines and memory impairment induced by lipopolysaccharide in rats. Cytokine.

[36] Marottoli FM, Katsumata Y, Koster KP, Thomas R, Fardo DW, Tai LM (2017). Peripheral Inflammation, Apolipoprotein E4, and Amyloid-beta Interact to Induce Cognitive and Cerebrovascular Dysfunction. ASN Neuro.

[37] Moussaieff A, Mechoulam R (2009). Boswellia resin: from religious ceremonies to medical uses; a review of in‐vitro, in‐vivo and clinical trials. J Pharm Pharmacol.

[38] Podda M V, Cocco S, Mastrodonato A, Fusco S, Leone L, Barbati S A, Colussi C, Ripoli C, Grassi C (2016). Anodal transcranial direct current stimulation boosts synaptic plasticity and memory in mice via epigenetic regulation of BDNF expression. Sci Rep.

[39] Quan N, Sundar SK, Weiss JM (1994). Induction of interleukin-1 in various brain regions after peripheral and central injections of lipopolysaccharide. J Neuroimmunol.

[40] Qurishi Y, Hamid A, Zargar M, Singh SK, Saxena AK (2010). Potential role of natural molecules in health and disease: Importance of boswellic acid. J Med Plants Res.

[41] Saavedra JM (2012). Angiotensin II AT1 receptor blockers as treatments for inflammatory brain disorders. Clin Sci.

[42] Sayed A, El Sayed N (2016). Co-administration of 3-acetyl-11-keto-beta-boswellic acid potentiates the protective effect of celecoxib in lipopolysaccharide-induced cognitive impairment in mice: possible implication of anti-inflammatory and antiglutamatergic pathways. J Mol Neurosci.

[43] Upaganlawar A, Ghule B (2009). Pharmacological activities of Boswellia serrata Roxb mini review. Ethnobot Leaflets..

[44] Weber C-C, Reising K, Müller WE, Schubert-Zsilavecz M, Abdel-Tawab M (2006). Modulation of Pgp function by boswellic acids. Planta medica.

[45] Zaker S, Beheshti S, Aghaie R, Noorbakhshnia M (2015). Effect of olibanum on a rat model of Alzheimer’s disease induced by intracerebroventricular injection of streptozotocin. J Physiol Pharmacol.

[46] Zheng WH, Quirion R (2004). Comparative signaling pathways of insulin‐like growth factor‐1 and brain‐derived neurotrophic factor in hippocampal neurons and the role of the PI3 kinase pathway in cell survival. J Neurochem.

